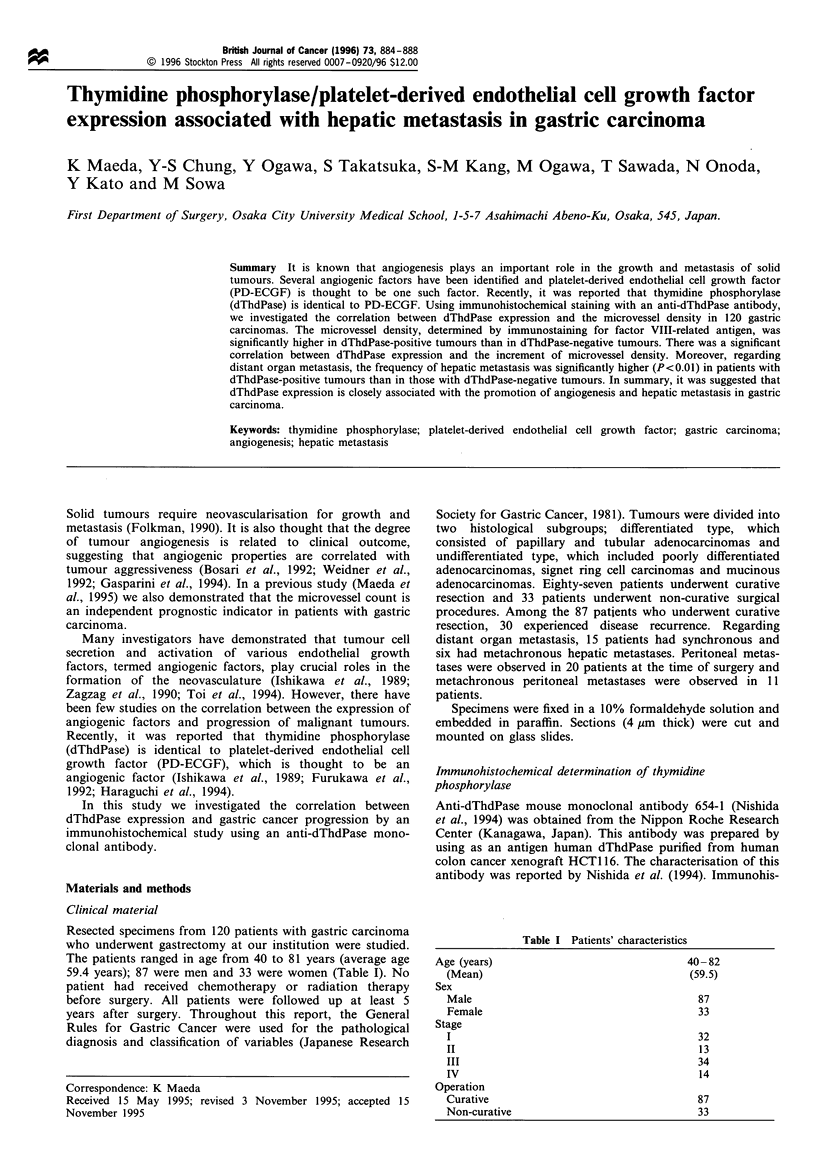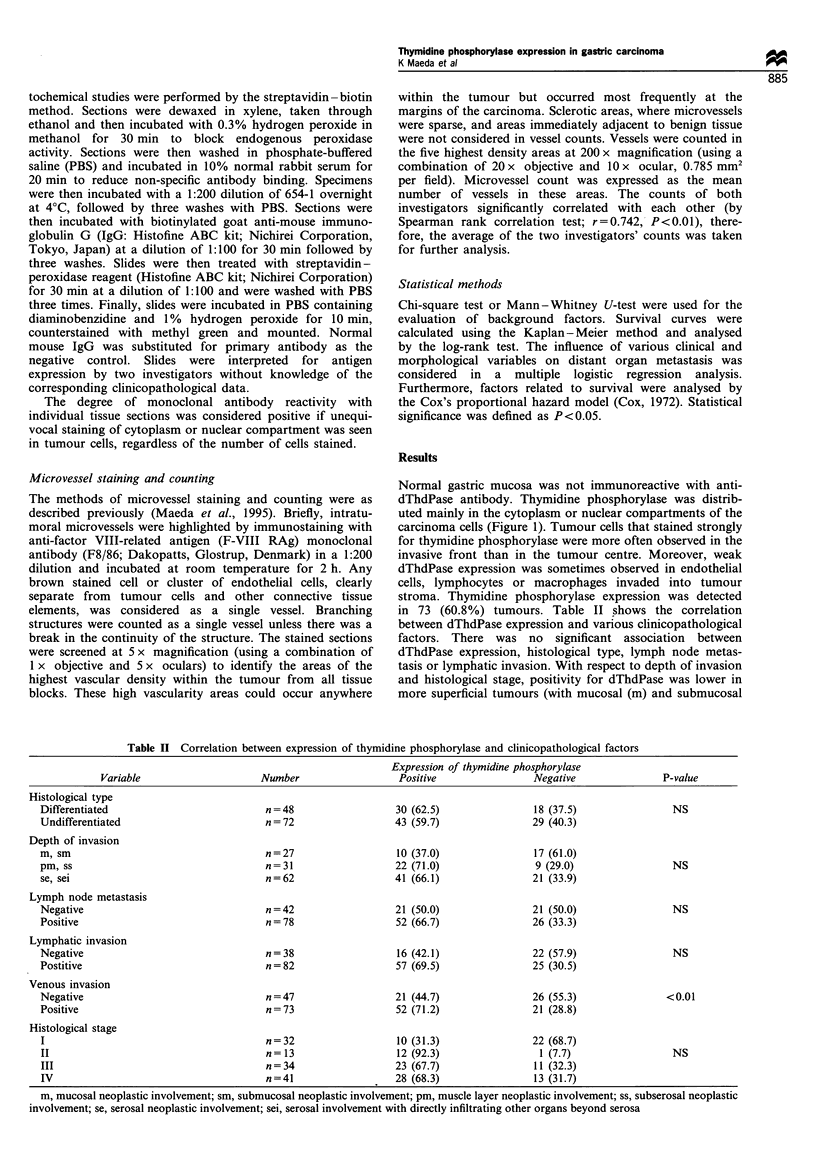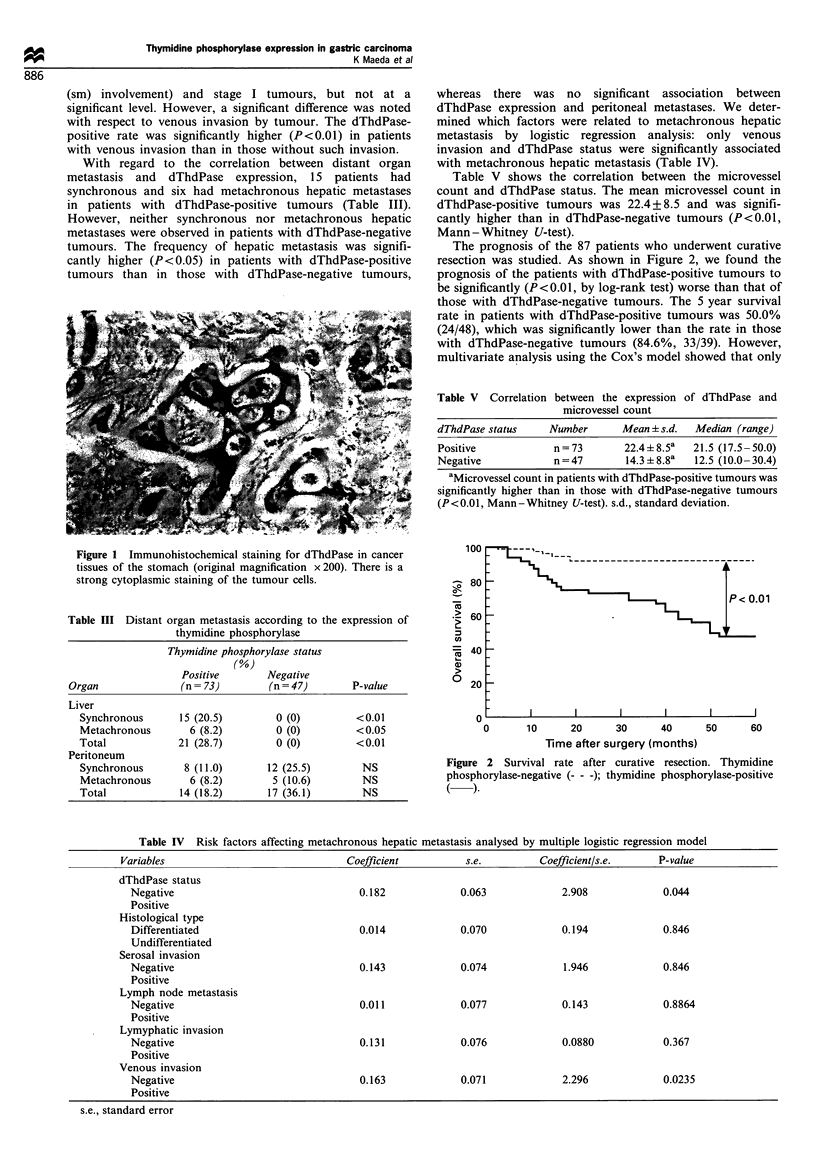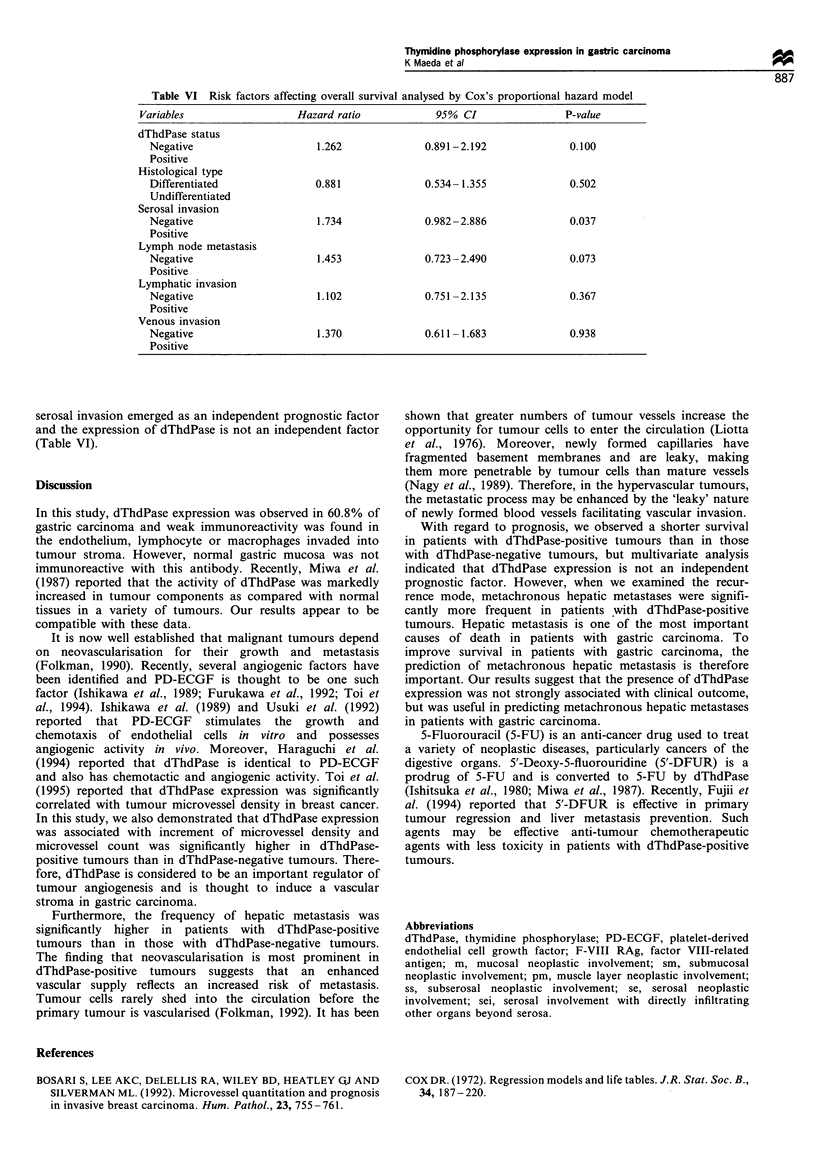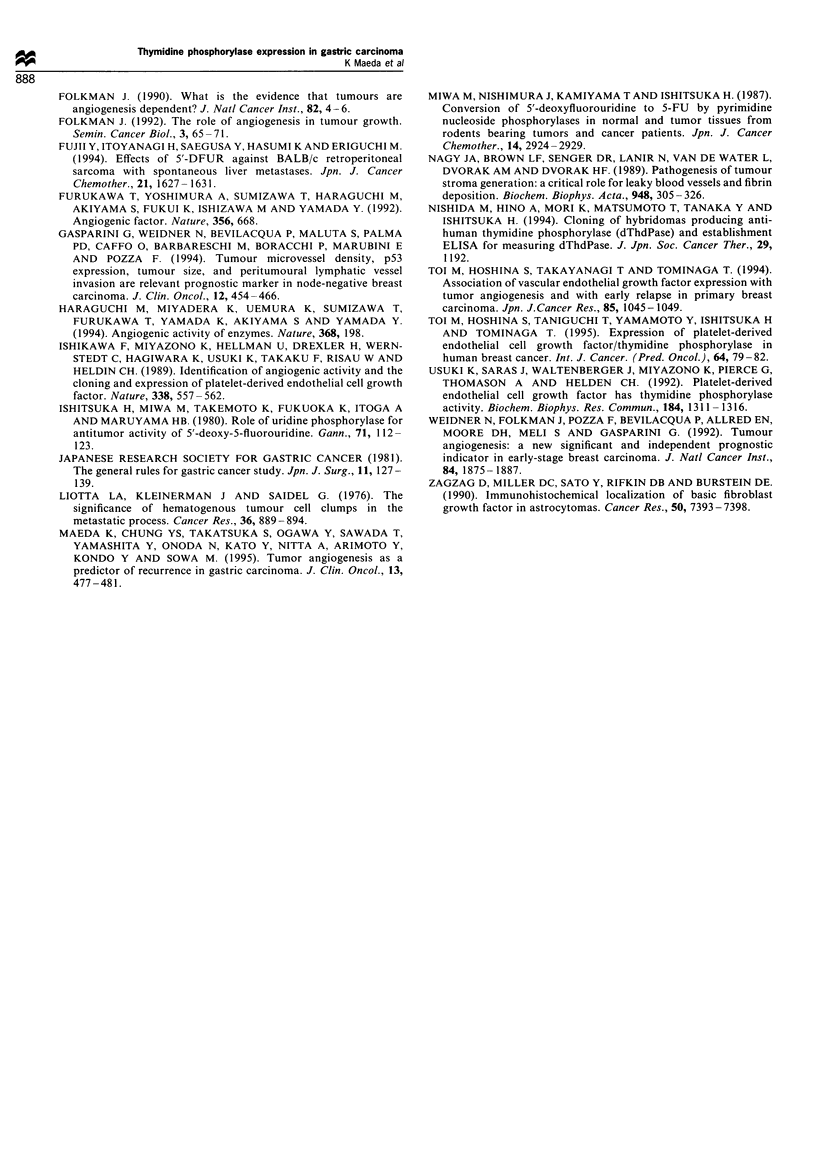# Thymidine phosphorylase/platelet-derived endothelial cell growth factor expression associated with hepatic metastasis in gastric carcinoma.

**DOI:** 10.1038/bjc.1996.177

**Published:** 1996-04

**Authors:** K. Maeda, Y. S. Chung, Y. Ogawa, S. Takatsuka, S. M. Kang, M. Ogawa, T. Sawada, N. Onoda, Y. Kato, M. Sowa

**Affiliations:** First Department of Surgery, Osaka City University Medical School, Japan.

## Abstract

**Images:**


British Journal of Cancer (1996) 73, 884-888
© 1996 Stockton Press All rights reserved 0007-0920/96 $12.00

### Thymidine phosphorylase/platelet-derived endothelial cell growth factor expression associated with hepatic metastasis in gastric carcinoma

K Maeda, Y-S Chung, Y Ogawa, S Takatsuka, S-M Kang, M Ogawa, T Sawada, N Onoda, Y Kato and M Sowa

*First Department of Surgery, Osaka City University Medical School, 1-5-7 Asahimachi Abeno-Ku, Osaka, 545, Japan.*

**Summary** It is known that angiogenesis plays an important role in the growth and metastasis of solid tumours. Several angiogenic factors have been identified and platelet-derived endothelial cell growth factor (PD-ECGF) is thought to be one such factor. Recently, it was reported that thymidine phosphorylase (dThdPase) is identical to PD-ECGF. Using immunohistochemical staining with an anti-dThdPase antibody, we investigated the correlation between dThdPase expression and the microvessel density in 120 gastric carcinomas. The microvessel density, determined by immunostaining for factor VIII-related antigen, was significantly higher in dThdPase-positive tumours than in dThdPase-negative tumours. There was a significant correlation between dThdPase expression and the increment of microvessel density. Moreover, regarding distant organ metastasis, the frequency of hepatic metastasis was significantly higher ($P<0.01$) in patients with dThdPase-positive tumours than in those with dThdPase-negative tumours. In summary, it was suggested that dThdPase expression is closely associated with the promotion of angiogenesis and hepatic metastasis in gastric carcinoma.

**Keywords:** thymidine phosphorylase; platelet-derived endothelial cell growth factor; gastric carcinoma; angiogenesis; hepatic metastasis

Solid tumours require neovascularisation for growth and metastasis (Folkman, 1990). It is also thought that the degree of tumour angiogenesis is related to clinical outcome, suggesting that angiogenic properties are correlated with tumour aggressiveness (Bosari *et al.*, 1992; Weidner *et al.*, 1992; Gasparini *et al.*, 1994). In a previous study (Maeda *et al.*, 1995) we also demonstrated that the microvessel count is an independent prognostic indicator in patients with gastric carcinoma.

Many investigators have demonstrated that tumour cell secretion and activation of various endothelial growth factors, termed angiogenic factors, play crucial roles in the formation of the neovasculature (Ishikawa *et al.*, 1989; Zagzag *et al.*, 1990; Toi *et al.*, 1994). However, there have been few studies on the correlation between the expression of angiogenic factors and progression of malignant tumours. Recently, it was reported that thymidine phosphorylase (dThdPase) is identical to platelet-derived endothelial cell growth factor (PD-ECGF), which is thought to be an angiogenic factor (Ishikawa *et al.*, 1989; Furukawa *et al.*, 1992; Haraguchi *et al.*, 1994).

In this study we investigated the correlation between dThdPase expression and gastric cancer progression by an immunohistochemical study using an anti-dThdPase monoclonal antibody.

### Materials and methods

### *Clinical material*

Resected specimens from 120 patients with gastric carcinoma who underwent gastrectomy at our institution were studied. The patients ranged in age from 40 to 81 years (average age 59.4 years); 87 were men and 33 were women (Table I). No patient had received chemotherapy or radiation therapy before surgery. All patients were followed up at least 5 years after surgery. Throughout this report, the General Rules for Gastric Cancer were used for the pathological diagnosis and classification of variables (Japanese Research Society for Gastric Cancer, 1981). Tumours were divided into two histological subgroups; differentiated type, which consisted of papillary and tubular adenocarcinomas and undifferentiated type, which included poorly differentiated adenocarcinomas, signet ring cell carcinomas and mucinous adenocarcinomas. Eighty-seven patients underwent curative resection and 33 patients underwent non-curative surgical procedures. Among the 87 patients who underwent curative resection, 30 experienced disease recurrence. Regarding distant organ metastasis, 15 patients had synchronous and six had metachronous hepatic metastases. Peritoneal metastases were observed in 20 patients at the time of surgery and metachronous peritoneal metastases were observed in 11 patients.

Specimens were fixed in a 10% formaldehyde solution and embedded in paraffin. Sections (4 $\mu$m thick) were cut and mounted on glass slides.

### *Immunohistochemical determination of thymidine phosphorylase*

Anti-dThdPase mouse monoclonal antibody 654-1 (Nishida *et al.*, 1994) was obtained from the Nippon Roche Research Center (Kanagawa, Japan). This antibody was prepared by using as an antigen human dThdPase purified from human colon cancer xenograft HCT116. The characterisation of this antibody was reported by Nishida *et al.* (1994). Immunohis-

**Table I** Patients' characteristics

| | |
|---|---|
| Age (years) | 40-82 |
| (Mean) | (59.5) |
| Sex | |
| Male | 87 |
| Female | 33 |
| Stage | |
| I | 32 |
| II | 13 |
| III | 34 |
| IV | 14 |
| Operation | |
| Curative | 87 |
| Non-curative | 33 |

Correspondence: K Maeda
Received 15 May 1995; revised 3 November 1995; accepted 15 November 1995

tochemical studies were performed by the streptavidin–biotin method. Sections were dewaxed in xylene, taken through ethanol and then incubated with 0.3% hydrogen peroxide in methanol for 30 min to block endogenous peroxidase activity. Sections were then washed in phosphate-buffered saline (PBS) and incubated in 10% normal rabbit serum for 20 min to reduce non-specific antibody binding. Specimens were then incubated with a 1:200 dilution of 654-1 overnight at 4°C, followed by three washes with PBS. Sections were then incubated with biotinylated goat anti-mouse immunoglobulin G (IgG: Histofine ABC kit; Nichirei Corporation, Tokyo, Japan) at a dilution of 1:100 for 30 min followed by three washes. Slides were then treated with streptavidin–peroxidase reagent (Histofine ABC kit; Nichirei Corporation) for 30 min at a dilution of 1:100 and were washed with PBS three times. Finally, slides were incubated in PBS containing diaminobenzidine and 1% hydrogen peroxide for 10 min, counterstained with methyl green and mounted. Normal mouse IgG was substituted for primary antibody as the negative control. Slides were interpreted for antigen expression by two investigators without knowledge of the corresponding clinicopathological data.

The degree of monoclonal antibody reactivity with individual tissue sections was considered positive if unequivocal staining of cytoplasm or nuclear compartment was seen in tumour cells, regardless of the number of cells stained.

### *Microvessel staining and counting*

The methods of microvessel staining and counting were as described previously (Maeda *et al*., 1995). Briefly, intratumoral microvessels were highlighted by immunostaining with anti-factor VIII-related antigen (F-VIII RAg) monoclonal antibody (F8/86; Dakopatts, Glostrup, Denmark) in a 1:200 dilution and incubated at room temperature for 2 h. Any brown stained cell or cluster of endothelial cells, clearly separate from tumour cells and other connective tissue elements, was considered as a single vessel. Branching structures were counted as a single vessel unless there was a break in the continuity of the structure. The stained sections were screened at 5 × magnification (using a combination of 1 × objective and 5 × oculars) to identify the areas of the highest vascular density within the tumour from all tissue blocks. These high vascularity areas could occur anywhere within the tumour but occurred most frequently at the margins of the carcinoma. Sclerotic areas, where microvessels were sparse, and areas immediately adjacent to benign tissue were not considered in vessel counts. Vessels were counted in the five highest density areas at 200 × magnification (using a combination of 20 × objective and 10 × ocular, 0.785 $mm^2$ per field). Microvessel count was expressed as the mean number of vessels in these areas. The counts of both investigators significantly correlated with each other (by Spearman rank correlation test; $r = 0.742$, $P < 0.01$), therefore, the average of the two investigators' counts was taken for further analysis.

### *Statistical methods*

Chi-square test or Mann–Whitney *U*-test were used for the evaluation of background factors. Survival curves were calculated using the Kaplan–Meier method and analysed by the log-rank test. The influence of various clinical and morphological variables on distant organ metastasis was considered in a multiple logistic regression analysis. Furthermore, factors related to survival were analysed by the Cox's proportional hazard model (Cox, 1972). Statistical significance was defined as $P < 0.05$.

### Results

Normal gastric mucosa was not immunoreactive with anti-dThdPase antibody. Thymidine phosphorylase was distributed mainly in the cytoplasm or nuclear compartments of the carcinoma cells (Figure 1). Tumour cells that stained strongly for thymidine phosphorylase were more often observed in the invasive front than in the tumour centre. Moreover, weak dThdPase expression was sometimes observed in endothelial cells, lymphocytes or macrophages invaded into tumour stroma. Thymidine phosphorylase expression was detected in 73 (60.8%) tumours. Table II shows the correlation between dThdPase expression and various clinicopathological factors. There was no significant association between dThdPase expression, histological type, lymph node metastasis or lymphatic invasion. With respect to depth of invasion and histological stage, positivity for dThdPase was lower in more superficial tumours (with mucosal (m) and submucosal

**Table II** Correlation between expression of thymidine phosphorylase and clinicopathological factors

| *Variable* | *Number* | *Expression of thymidine phosphorylase* Positive | Negative | P-*value* |
|---|---|---|---|---|
| Histological type | | | | |
| Differentiated | $n=48$ | 30 (62.5) | 18 (37.5) | NS |
| Undifferentiated | $n=72$ | 43 (59.7) | 29 (40.3) | |
| Depth of invasion | | | | |
| m, sm | $n=27$ | 10 (37.0) | 17 (61.0) | |
| pm, ss | $n=31$ | 22 (71.0) | 9 (29.0) | NS |
| se, sei | $n=62$ | 41 (66.1) | 21 (33.9) | |
| Lymph node metastasis | | | | |
| Negative | $n=42$ | 21 (50.0) | 21 (50.0) | NS |
| Positive | $n=78$ | 52 (66.7) | 26 (33.3) | |
| Lymphatic invasion | | | | |
| Negative | $n=38$ | 16 (42.1) | 22 (57.9) | NS |
| Postitive | $n=82$ | 57 (69.5) | 25 (30.5) | |
| Venous invasion | | | | |
| Negative | $n=47$ | 21 (44.7) | 26 (55.3) | <0.01 |
| Positive | $n=73$ | 52 (71.2) | 21 (28.8) | |
| Histological stage | | | | |
| I | $n=32$ | 10 (31.3) | 22 (68.7) | |
| II | $n=13$ | 12 (92.3) | 1 (7.7) | NS |
| III | $n=34$ | 23 (67.7) | 11 (32.3) | |
| IV | $n=41$ | 28 (68.3) | 13 (31.7) | |

m, mucosal neoplastic involvement; sm, submucosal neoplastic involvement; pm, muscle layer neoplastic involvement; ss, subserosal neoplastic involvement; se, serosal neoplastic involvement; sei, serosal involvement with directly infiltrating other organs beyond serosa

(sm) involvement) and stage I tumours, but not at a significant level. However, a significant difference was noted with respect to venous invasion by tumour. The dThdPase-positive rate was significantly higher ($P<0.01$) in patients with venous invasion than in those without such invasion.

With regard to the correlation between distant organ metastasis and dThdPase expression, 15 patients had synchronous and six had metachronous hepatic metastases in patients with dThdPase-positive tumours (Table III). However, neither synchronous nor metachronous hepatic metastases were observed in patients with dThdPase-negative tumours. The frequency of hepatic metastasis was significantly higher ($P<0.05$) in patients with dThdPase-positive tumours than in those with dThdPase-negative tumours, whereas there was no significant association between dThdPase expression and peritoneal metastases. We determined which factors were related to metachronous hepatic metastasis by logistic regression analysis: only venous invasion and dThdPase status were significantly associated with metachronous hepatic metastasis (Table IV).

Table V shows the correlation between the microvessel count and dThdPase status. The mean microvessel count in dThdPase-positive tumours was 22.4±8.5 and was significantly higher than in dThdPase-negative tumours ($P<0.01$, Mann–Whitney $U$-test).

The prognosis of the 87 patients who underwent curative resection was studied. As shown in Figure 2, we found the prognosis of the patients with dThdPase-positive tumours to be significantly ($P<0.01$, by log-rank test) worse than that of those with dThdPase-negative tumours. The 5 year survival rate in patients with dThdPase-positive tumours was 50.0% (24/48), which was significantly lower than the rate in those with dThdPase-negative tumours (84.6%, 33/39). However, multivariate analysis using the Cox's model showed that only

**Figure 1** Immunohistochemical staining for dThdPase in cancer tissues of the stomach (original magnification ×200). There is a strong cytoplasmic staining of the tumour cells.

**Table III** Distant organ metastasis according to the expression of thymidine phosphorylase

| Organ | Thymidine phosphorylase status (%) Positive (n=73) | Negative (n=47) | P-value |
|---|---|---|---|
| Liver | | | |
| Synchronous | 15 (20.5) | 0 (0) | <0.01 |
| Metachronous | 6 (8.2) | 0 (0) | <0.05 |
| Total | 21 (28.7) | 0 (0) | <0.01 |
| Peritoneum | | | |
| Synchronous | 8 (11.0) | 12 (25.5) | NS |
| Metachronous | 6 (8.2) | 5 (10.6) | NS |
| Total | 14 (18.2) | 17 (36.1) | NS |

**Table V** Correlation between the expression of dThdPase and microvessel count

| dThdPase status | Number | Mean±s.d. | Median (range) |
|---|---|---|---|
| Positive | n=73 | 22.4±8.5[a] | 21.5 (17.5–50.0) |
| Negative | n=47 | 14.3±8.8[a] | 12.5 (10.0–30.4) |

[a]Microvessel count in patients with dThdPase-positive tumours was significantly higher than in those with dThdPase-negative tumours ($P<0.01$, Mann–Whitney $U$-test). s.d., standard deviation.

**Figure 2** Survival rate after curative resection. Thymidine phosphorylase-negative (- - -); thymidine phosphorylase-positive (——).

**Table IV** Risk factors affecting metachronous hepatic metastasis analysed by multiple logistic regression model

| Variables | Coefficient | s.e. | Coefficient/s.e. | P-value |
|---|---|---|---|---|
| dThdPase status | | | | |
| Negative | 0.182 | 0.063 | 2.908 | 0.044 |
| Positive | | | | |
| Histological type | | | | |
| Differentiated | 0.014 | 0.070 | 0.194 | 0.846 |
| Undifferentiated | | | | |
| Serosal invasion | | | | |
| Negative | 0.143 | 0.074 | 1.946 | 0.846 |
| Positive | | | | |
| Lymph node metastasis | | | | |
| Negative | 0.011 | 0.077 | 0.143 | 0.8864 |
| Positive | | | | |
| Lymyphatic invasion | | | | |
| Negative | 0.131 | 0.076 | 0.0880 | 0.367 |
| Positive | | | | |
| Venous invasion | | | | |
| Negative | 0.163 | 0.071 | 2.296 | 0.0235 |
| Positive | | | | |

s.e., standard error

**Table VI** Risk factors affecting overall survival analysed by Cox's proportional hazard model

| *Variables* | *Hazard ratio* | *95% CI* | P-*value* |
|---|---|---|---|
| dThdPase status | | | |
| Negative | 1.262 | 0.891-2.192 | 0.100 |
| Positive | | | |
| Histological type | | | |
| Differentiated | 0.881 | 0.534-1.355 | 0.502 |
| Undifferentiated | | | |
| Serosal invasion | | | |
| Negative | 1.734 | 0.982-2.886 | 0.037 |
| Positive | | | |
| Lymph node metastasis | | | |
| Negative | 1.453 | 0.723-2.490 | 0.073 |
| Positive | | | |
| Lymphatic invasion | | | |
| Negative | 1.102 | 0.751-2.135 | 0.367 |
| Positive | | | |
| Venous invasion | | | |
| Negative | 1.370 | 0.611-1.683 | 0.938 |
| Positive | | | |

serosal invasion emerged as an independent prognostic factor and the expression of dThdPase is not an independent factor (Table VI).

### Discussion

In this study, dThdPase expression was observed in 60.8% of gastric carcinoma and weak immunoreactivity was found in the endothelium, lymphocyte or macrophages invaded into tumour stroma. However, normal gastric mucosa was not immunoreactive with this antibody. Recently, Miwa *et al.* (1987) reported that the activity of dThdPase was markedly increased in tumour components as compared with normal tissues in a variety of tumours. Our results appear to be compatible with these data.

It is now well established that malignant tumours depend on neovascularisation for their growth and metastasis (Folkman, 1990). Recently, several angiogenic factors have been identified and PD-ECGF is thought to be one such factor (Ishikawa *et al.*, 1989; Furukawa *et al.*, 1992; Toi *et al.*, 1994). Ishikawa *et al.* (1989) and Usuki *et al.* (1992) reported that PD-ECGF stimulates the growth and chemotaxis of endothelial cells *in vitro* and possesses angiogenic activity *in vivo*. Moreover, Haraguchi *et al.* (1994) reported that dThdPase is identical to PD-ECGF and also has chemotactic and angiogenic activity. Toi *et al.* (1995) reported that dThdPase expression was significantly correlated with tumour microvessel density in breast cancer. In this study, we also demonstrated that dThdPase expression was associated with increment of microvessel density and microvessel count was significantly higher in dThdPase-positive tumours than in dThdPase-negative tumours. Therefore, dThdPase is considered to be an important regulator of tumour angiogenesis and is thought to induce a vascular stroma in gastric carcinoma.

Furthermore, the frequency of hepatic metastasis was significantly higher in patients with dThdPase-positive tumours than in those with dThdPase-negative tumours. The finding that neovascularisation is most prominent in dThdPase-positive tumours suggests that an enhanced vascular supply reflects an increased risk of metastasis. Tumour cells rarely shed into the circulation before the primary tumour is vascularised (Folkman, 1992). It has been shown that greater numbers of tumour vessels increase the opportunity for tumour cells to enter the circulation (Liotta *et al.*, 1976). Moreover, newly formed capillaries have fragmented basement membranes and are leaky, making them more penetrable by tumour cells than mature vessels (Nagy *et al.*, 1989). Therefore, in the hypervascular tumours, the metastatic process may be enhanced by the 'leaky' nature of newly formed blood vessels facilitating vascular invasion.

With regard to prognosis, we observed a shorter survival in patients with dThdPase-positive tumours than in those with dThdPase-negative tumours, but multivariate analysis indicated that dThdPase expression is not an independent prognostic factor. However, when we examined the recurrence mode, metachronous hepatic metastases were significantly more frequent in patients with dThdPase-positive tumours. Hepatic metastasis is one of the most important causes of death in patients with gastric carcinoma. To improve survival in patients with gastric carcinoma, the prediction of metachronous hepatic metastasis is therefore important. Our results suggest that the presence of dThdPase expression was not strongly associated with clinical outcome, but was useful in predicting metachronous hepatic metastases in patients with gastric carcinoma.

5-Fluorouracil (5-FU) is an anti-cancer drug used to treat a variety of neoplastic diseases, particularly cancers of the digestive organs. 5′-Deoxy-5-fluorouridine (5′-DFUR) is a prodrug of 5-FU and is converted to 5-FU by dThdPase (Ishitsuka *et al.*, 1980; Miwa *et al.*, 1987). Recently, Fujii *et al.* (1994) reported that 5′-DFUR is effective in primary tumour regression and liver metastasis prevention. Such agents may be effective anti-tumour chemotherapeutic agents with less toxicity in patients with dThdPase-positive tumours.

### Abbreviations dThdPase, thymidine phosphorylase; PD-ECGF, platelet-derived endothelial cell growth factor; F-VIII RAg, factor VIII-related antigen; m, mucosal neoplastic involvement; sm, submucosal neoplastic involvement; pm, muscle layer neoplastic involvement; ss, subserosal neoplastic involvement; se, serosal neoplastic involvement; sei, serosal involvement with directly infiltrating other organs beyond serosa.

### References

BOSARI S, LEE AKC, DELELLIS RA, WILEY BD, HEATLEY GJ AND SILVERMAN ML. (1992). Microvessel quantitation and prognosis in invasive breast carcinoma. *Hum. Pathol.*, **23**, 755-761.

COX DR. (1972). Regression models and life tables. *J.R. Stat. Soc. B.*, **34**, 187-220.

FOLKMAN J. (1990). What is the evidence that tumours are angiogenesis dependent? *J. Natl Cancer Inst.*, **82**, 4-6.

FOLKMAN J. (1992). The role of angiogenesis in tumour growth. *Semin. Cancer Biol.*, **3**, 65-71.

FUJII Y, ITOYANAGI H, SAEGUSA Y, HASUMI K AND ERIGUCHI M. (1994). Effects of 5'-DFUR against BALB/c retroperitoneal sarcoma with spontaneous liver metastases. *Jpn. J. Cancer Chemother.*, **21**, 1627-1631.

FURUKAWA T, YOSHIMURA A, SUMIZAWA T, HARAGUCHI M, AKIYAMA S, FUKUI K, ISHIZAWA M AND YAMADA Y. (1992). Angiogenic factor. *Nature*, **356**, 668.

GASPARINI G, WEIDNER N, BEVILACQUA P, MALUTA S, PALMA PD, CAFFO O, BARBARESCHI M, BORACCHI P, MARUBINI E AND POZZA F. (1994). Tumour microvessel density, p53 expression, tumour size, and peritumoural lymphatic vessel invasion are relevant prognostic marker in node-negative breast carcinoma. *J. Clin. Oncol.*, **12**, 454-466.

HARAGUCHI M, MIYADERA K, UEMURA K, SUMIZAWA T, FURUKAWA T, YAMADA K, AKIYAMA S AND YAMADA Y. (1994). Angiogenic activity of enzymes. *Nature*, **368**, 198.

ISHIKAWA F, MIYAZONO K, HELLMAN U, DREXLER H, WERNSTEDT C, HAGIWARA K, USUKI K, TAKAKU F, RISAU W AND HELDIN CH. (1989). Identification of angiogenic activity and the cloning and expression of platelet-derived endothelial cell growth factor. *Nature*, **338**, 557-562.

ISHITSUKA H, MIWA M, TAKEMOTO K, FUKUOKA K, ITOGA A AND MARUYAMA HB. (1980). Role of uridine phosphorylase for antitumor activity of 5'-deoxy-5-fluorouridine. *Gann.*, **71**, 112-123.

JAPANESE RESEARCH SOCIETY FOR GASTRIC CANCER (1981). The general rules for gastric cancer study. *Jpn. J. Surg.*, **11**, 127-139.

LIOTTA LA, KLEINERMAN J AND SAIDEL G. (1976). The significance of hematogenous tumour cell clumps in the metastatic process. *Cancer Res.*, **36**, 889-894.

MAEDA K, CHUNG YS, TAKATSUKA S, OGAWA Y, SAWADA T, YAMASHITA Y, ONODA N, KATO Y, NITTA A, ARIMOTO Y, KONDO Y AND SOWA M. (1995). Tumor angiogenesis as a predictor of recurrence in gastric carcinoma. *J. Clin. Oncol.*, **13**, 477-481.

MIWA M, NISHIMURA J, KAMIYAMA T AND ISHITSUKA H. (1987). Conversion of 5'-deoxyfluorouridine to 5-FU by pyrimidine nucleoside phosphorylases in normal and tumor tissues from rodents bearing tumors and cancer patients. *Jpn. J. Cancer Chemother.*, **14**, 2924-2929.

NAGY JA, BROWN LF, SENGER DR, LANIR N, VAN DE WATER L, DVORAK AM AND DVORAK HF. (1989). Pathogenesis of tumour stroma generation: a critical role for leaky blood vessels and fibrin deposition. *Biochem. Biophys. Acta.*, **948**, 305-326.

NISHIDA M, HINO A, MORI K, MATSUMOTO T, TANAKA Y AND ISHITSUKA H. (1994). Cloning of hybridomas producing anti-human thymidine phosphorylase (dThdPase) and establishment ELISA for measuring dThdPase. *J. Jpn. Soc. Cancer Ther.*, **29**, 1192.

TOI M, HOSHINA S, TAKAYANAGI T AND TOMINAGA T. (1994). Association of vascular endothelial growth factor expression with tumor angiogenesis and with early relapse in primary breast carcinoma. *Jpn. J.Cancer Res.*, **85**, 1045-1049.

TOI M, HOSHINA S, TANIGUCHI T, YAMAMOTO Y, ISHITSUKA H AND TOMINAGA T. (1995). Expression of platelet-derived endothelial cell growth factor/thymidine phosphorylase in human breast cancer. *Int. J. Cancer. (Pred. Oncol.)*, **64**, 79-82.

USUKI K, SARAS J, WALTENBERGER J, MIYAZONO K, PIERCE G, THOMASON A AND HELDEN CH. (1992). Platelet-derived endothelial cell growth factor has thymidine phosphorylase activity. *Biochem. Biophys. Res. Commun.*, **184**, 1311-1316.

WEIDNER N, FOLKMAN J, POZZA F, BEVILACQUA P, ALLRED EN, MOORE DH, MELI S AND GASPARINI G. (1992). Tumour angiogenesis: a new significant and independent prognostic indicator in early-stage breast carcinoma. *J. Natl Cancer Inst.*, **84**, 1875-1887.

ZAGZAG D, MILLER DC, SATO Y, RIFKIN DB AND BURSTEIN DE. (1990). Immunohistochemical localization of basic fibroblast growth factor in astrocytomas. *Cancer Res.*, **50**, 7393-7398.